# Supplementary material for: Impact of vaccination education in cardiac rehabilitation on attitudes and knowledge
Source: PLOS Glob Public Health. 2024 Mar 8;4(3):e0002610. doi: 10.1371/journal.pgph.0002610 (PMC10923443; doi:10.1371/journal.pgph.0002610)
Supplement: Attachment — Submitted filename: CR & Vaccinations_MSP_brief report_vPLOSGPH_Resp2Ed.docx [file pgph.0002610.s002.docx]

Dear Dr. Wagner:

Thank you for considering our paper for review. We have addressed your important points.

1 - could you better explain Hake's gain and/or reference some resources? I'm not familiar with it, and after searching around, I do think it could be applicable here. Yet, I think the average readership for this journal would not quite understand it, so providing that info could be useful.

RESPONSE: A MORE EXPLANATORY CITATION HAS BEEN ADDED.

2 - you have a lot of outcomes, I believe around 12 in Table 1. Ideally you can do some sort of correction for multiple p-values. Alternatively, you could pre-specify in an aim what is your main outcome in a confirmatory analysis, and then put everything else in a separate table as an explanatory analysis (with the exploratory analyses correcting for multiple p-values.

RESPONSE. A BONFERRONI CORRECTION HAS BEEN APPLIED. THIS IS DENOTED IN THE METHODS, RESULTS AND TABLE.

Sincerely,

Prof. Grace on behalf of the authors
