# Supplementary material for: Impact of vaccination education in cardiac rehabilitation on attitudes and knowledge
Source: PLOS Glob Public Health. 2024 Mar 8;4(3):e0002610. doi: 10.1371/journal.pgph.0002610 (PMC10923443; doi:10.1371/journal.pgph.0002610)
Supplement: Attachment — Submitted filename: Response to Reviewers_CR Vaccinations.docx [file pgph.0002610.s003.docx]

PGPH-D-23-02047R1, entitled "Impact of Vaccination Education in Cardiac Rehabilitation on Attitudes and Knowledge"

January 31, 2024

Dear Dr. Abram L. Wagner,

Thank you for the opportunity to submit a revision of the above-titled manuscript. Please find below a detailed response to the editorial committee and to each reviewer comment. Corresponding edits are tracked in the manuscript.

**Additional Editor Comments:**

1. In the introduction could you mention lack of access to vaccination in cardiac facilities? Presumably one barrier is that as individuals go to rehab, there is not a convenient place to access vaccination at those locations. (Feel free to disagree)

*RESPONSE:* Good point. Added

2. Can you confirm details about the intervention. It was forty (40) minutes long? Was that just about vaccines? Or were there other components to that 40 minute talk? If it really was a 40 minute talk on vaccines, possibly worth mentioning a limitation being the substantial burden of time this intervention requires.

*RESPONSE:* Patient education sessions in cardiac rehab are on average 30 minutes, and then patients often like to discuss and ask questions so it can go to 45 minutes based on patient preference! But agreed, given the volume of information CR programs need to cover, this is lengthy for one topic. The session was facilitated through a 21-slide powerpoint presentation, with question and answer at the end. More detail has been added abou the session and length has been added as a limitation.

3. You could mention in the discussion that this research is in line with vaccine studies in general which have shown provider recommendations to be one of the strongest predictors of vaccination (many such references exist - but possibly you could find a review related to vaccination of individuals with chronic disease)

RESPONSE: Good point. Also done

**Reviewer Comments:**

Reviewer #1:

4. Introduction

Line 49 - "Respiratory infections such influenza and pneumococcus -- and more recently coronavirus disease (COVID-19) – are associated with poorer outcomes in CVD patients." Can the authors provide references for the statement?

*RESPONSE:* Done

5. Introduction

Line 55 - "Accordingly, our CR program has augmented our education, to raise awareness and educate cardiovascular patients about both influenza and COVID-19 vaccines. The aim of this study was evaluate the education efficacy in changing vaccination knowledge and attitudes." The sentences need to be improved, please provide more details for the objectives of the study.

*RESPONSE:* Thank you for pointing out we made a grammatical error. This has now been corrected and the sentence improved for clarity.

6. Methods

Line 68 "The questionnaire items were developed by the authors, and piloted in several patients." Please provide references if other standardised questionnaires have been referred to and served as a source for the content.

*RESPONSE:* They were not.

7. Results

Line 85 "Eighty-five participants consented" could the authors also provide the total number of CR patients approached for the study? It will provide the reader the consent rate.

*RESPONSE:* 117 and the consent rate is now stated as well. We do mention the convenience sample as a limitation in the Discussion section.

8. Results

Please provide a Table of Basic Demographic of the participants.

*RESPONSE:* This has been added. The only other available demographic variable is neighbourhood, but that would not provide much information for the reader, particularly considering most lived in the same neighbourhood for proximity to the CR centre.

9. Results

Please provide a copy of the questionnaire.

*RESPONSE:* We have uploaded it, but please bear in mind that it is in Spanish.

10. Results

Please provide a statement on data availability. For the attached Excel file, what are in the columns p4 and p5? Are they patients identifiers? Dat should be de- identified for information shared in public domains.

*RESPONSE:* A data availability statement is provided as follows: “Data are available with publication as supporting information file”. There are no patient identifiers. They are occupation and city. Occupation has been removed out of an abundance of caution and the file replaced in the online system.

We hope you find these revisions satisfactory for publication of our work in your esteemed journal. Please let us know if we can provide anything further.

Sincerely,

The Authors
